# Tailored Treatment of Acute Ischemic Stroke: A Narrative Review of Evidence-Based Strategies by Imaging Type and Thrombectomy Availability

**DOI:** 10.3390/medicina61091700

**Published:** 2025-09-18

**Authors:** Odysseas Kargiotis, Klearchos Psychogios, Apostolos Safouris, Elisabeth Chroni, Petros Zampakis, Vasileios Panagiotopoulos, John Ellul, Georgios Tsivgoulis

**Affiliations:** 1 Department of Neurology, General University Hospital of Patras, School of Medicine, University of Patras, 26504 Patras, Greece; echroni@yahoo.com (E.C.); ellul@upatras.gr (J.E.); 2 Stroke Unit, Metropolitan Hospital, 18547 Piraeus, Greece; apsychoyio@yahoo.gr (K.P.); safouris@yahoo.com (A.S.); 3 Second Department of Neurology, “Attikon” University Hospital, School of Medicine, National & Kapodistrian University of Athens, Rimini 1, Chaidari, 12462 Athens, Greece; tsivgoulisgiorg@yahoo.gr; 4 Department of Radiology, General University Hospital of Patras, 26504 Patras, Greece; pzampakis@gmail.com; 5 Department of Neurosurgery, General University Hospital of Patras, 26504 Patras, Greece; panagiotopoulos2000@yahoo.com

**Keywords:** acute ischemic stroke, intravenous thrombolysis, mechanical thrombectomy, advanced imaging

## Abstract

Stroke is a leading cause of disability and the second cause of death in adults. Moreover, the incidence of stroke is continuously rising. Acute reperfusion therapies (ARTs) have revolutionized stroke medicine and have altered the natural course of acute ischemic stroke. However, these treatments are ultimately offered to only a minority of acute ischemic stroke (AIS) patients, primarily due to delays in presentation. The use of advanced imaging has partially increased eligibility for ART; nevertheless, a large proportion of AIS patients remain untreated. In addition, many stroke centers lack readily available advanced imaging, sometimes lacking even computed tomography angiography. In these settings, several recent studies have sought to simplify the imaging prerequisites and criteria for the administration of ARTs. In this review, we discuss the possible treatment options for AIS patients presenting in different time points, according to type of imaging availability and mechanical thrombectomy availability. Our aim is to provide evidence-based recommendations, but also to analyze emerging data supporting the individualized, off-label use of ART without the aid of advanced imaging.

## 1. Introduction

Stroke is a leading cause of disability and death in adults [1]. During the recent decades there has been a striking 70% increase in the incidence of new stroke cases worldwide [2]. In 2021, there were over 7 million deaths from stroke globally, and this number is expected to increase further as the population ages [2]. Approximately 80% of strokes are of the ischemic type and recent years have witnessed significant breakthroughs in their acute treatment [3]. Indeed, the introduction of acute reperfusion therapies (ARTs) has revolutionized stroke medicine and altered the natural course of acute ischemic stroke (AIS) [4,5]. This is particularly relevant for large vessel occlusion (LVO)-related acute ischemic stroke (LVO-AIS), comprising 30% of all AIS cases, where reperfusion treatments, intravenous thrombolysis (IVT), and endovascular thrombectomy (EVT) are potentially applicable [6].

However, only a fraction of acute ischemic stroke (AIS) patients benefit from these powerful treatments. A recently published study reported a global incidence of AIS of 101.3 (91–113.6) per 100,000, with rates of IVT < 5% and fewer than 100,000 EVTs performed in total. Obviously, there is a continuous increase in the rates of acute reperfusion treatments. For instance, in the USA, rates of IVT increased from 6.5% in 2004 to 11% in 2016, and EVT increased from 0.1% in 2004 to 2.4% in 2016 [7]. In Europe, 17.14% (95% CI, 12.98–21.30) of AIS patients received IVT, and 6.91% (95% CI, 5.15–8.67) underwent EVT in 2019 [8]. Rates vary considerably between countries; IVT rates range from 52.66% to less than 0.1% of AIS patients, whereas those of EVT range between 21.8% and 0%, reflecting the large inequalities in acute stroke care between high- and low-income countries [8].

Therefore, even in countries with adequate resources and a high level of acute stroke care facilities, IVT and EVT are currently offered to the minority of AIS patients. The main reason for not delivering IVT is the narrow therapeutic time window. Consequently, more than one quarter of patients present with an unknown stroke onset time or with symptoms upon awakening [9]. A previously published retrospective study from the Acute Stroke Registry and Analysis of Lausanne found that 66.3% of patients do not receive IVT because of presentation outside the indicated time window or because of unknown time onset. Imaging criteria exclude only a minority of AIS patients (4.6%) from IVT [10]. In contrast, a prospective population-based multicenter study from Catalonia, Spain (Codi Ictus Catalunya registry), reported that IVT was more often withheld due to neuroimaging criteria—such as a low Alberta Stroke Program Early CT Score (ASPECTS) or absence of perfusion mismatch—in 10.5% of LVO-AIS cases, mild symptoms in 7%, and only in 0.7% due to presentation beyond 8 h from symptom onset [11].

In recent years, several completed or ongoing studies have been designed with the aim to extend the eligibility of AIS patients for reperfusion therapies, by either extending the time window and/or utilizing advanced imaging to identify patients with delayed time presentation, unknown time onset, large ischemic core, or mild or severe symptoms, who might benefit from ARTs [12,13]. In this narrative review, we present evidence-based recommendations for guiding individualized IVT and EVT decisions based on imaging or EVT availability and clinical presentation. A comprehensive search of the literature on intravenous thrombolysis and mechanical thrombectomy in acute ischemic stroke was performed using MEDLINE (PubMed), covering studies published from inception to August 2025. The search strategy included the term “acute ischemic stroke” combined with the terms “intravenous thrombolysis”, “mechanical thrombectomy”, “extended time window”, “unknown time onset”, “wake-up”, “advanced imaging”, “posterior circulation stroke”, “basilar artery occlusion”, “large core “, “distal vessel occlusion”, “medium vessel occlusion”, and “minor stroke and large vessel occlusion”.

We considered peer-reviewed publications written in English, and eligible study types included randomized controlled trials, observational studies, systematic reviews and meta-analyses, narrative reviews, and clinical practice guidelines. Studies that were thematically unrelated, conducted in animals, or categorized as editorials, commentaries, case reports, or preprints were excluded. We finally selected articles relevant to each section (time windows, imaging availability) with an initial priority to randomized controlled trials, clinical practice guidelines, and meta-analyses, followed by large multicenter observational studies and systematic or narrative reviews.

## 2. Relevant Section

### 2.1. Patients Presenting Within 4.5 h in a Non-Thrombectomy-Capable Center (Figure 1)

In patients presenting within the 4.5 h time window with disabling symptoms, IVT can be administered based solely on plain brain computed tomography (CT) [14]. In the case where no CT angiography (CTA) is available, treating physicians may use established stroke scales for LVO-AIS prediction, or, simply, the total National Institutes of Health Stroke Scale (NIHSS) score with a threshold of 7, which has been shown to have a sensitivity of 81.0%, specificity of 76.6%, positive predicting value of 84.2%, negative predictive value of 72.4%, and accuracy of 79.3% for predicting LVO-AIS. Incorporating the presence of ‘cortical’ symptoms—such as aphasia, neglect, hemianopia, and gaze deviation—as well as upper arm weakness, may improve diagnostic accuracy [15]. Immediate transfer of these patients, as well as those with a clinical suspicion of basilar artery occlusion, to a thrombectomy-capable center is warranted. The transition from alteplase to tenecteplase as the thrombolytic of choice—given as a bolus injection instead of a 1 h infusion, due to its longer half-life—facilitates the workflow of patients, diminishes time delays, and minimizes dosing errors. These advantages, derived from the adoption of tenecteplase as the thrombolytic of choice, may prove particularly valuable in resource-limited settings, potentially reducing the cost of AIS management. Moreover, tenecteplase, compared to alteplase, has an increased resistance to the plasminogen activator inhibitor 1 and a higher fibrin specificity resulting in an improved safety profile and reduced systemic bleeding [16,17,18].

Alternatively, treating physicians may base the decision to transfer the patient with LVO-AIS on a total NIHSS score cut-off of ≥5. Recent research has shown that, in such cases, the best medical treatment is equivalent to EVT in the presence of LVO; therefore, transfer may be withheld [19]. Finally, in patients with minor AIS and non-disabling symptoms, the superiority of dual antiplatelet therapy over IVT has been shown in a recent network meta-analysis [20].

### 2.2. Patients with LVO Presenting Within 4.5 h in a Thrombectomy-Capable Center (Figure 2)

Patients with LVO-related AIS, presenting directly to a thrombectomy-capable center, are candidates for both ARTs. Given the relatively low rates of successful recanalization with IVT in AIS due to LVO, it has been argued that IVT may be omitted in favor of proceeding directly to endovascular thrombectomy (EVT), which represents the more effective reperfusion strategy in this setting [21,22]. The rationale for this approach includes concerns about a potentially higher risk of systemic and intracranial hemorrhage with bridging therapy (IVT followed by EVT), the restriction of antiplatelet use in cases requiring adjunctive stenting, the occurrence of orolingual angioedema, and the risk of clot fragmentation with subsequent distal embolization [22]. Nevertheless, a previous meta-analysis of data from observational studies found improved functional outcomes with bridging therapy compared to direct EVT, and, similarly, a study-level meta-analysis of RCTs failed to show the non-inferiority of direct EVT versus bridging therapy (IVT plus EVT) based on non-inferiority margins of both 1.3% and 5.0% [23,24]. Thus, IVT prior to EVT is recommended in settings where patients present to both thrombectomy-capable and non-capable centers [24]. This may be even more critical in the new era of tenecteplase use, given its rapid administration and superior recanalization efficacy [25]. This argument is further strengthened by the results of the recently published BRIDGE-TNK trial, showing that, among AIS patients, and due to LVO, who had presented within 4.5 h after onset, the percentage of patients with functional independence at 90 days was higher with intravenous tenecteplase plus EVT than with endovascular thrombectomy alone [26].

### 2.3. Patients Presenting >4.5 h from Symptom Onset or with Unknown Time of Onset in a Non-Thrombectomy-Capable Center and Without Advanced Imaging (Figure 3)

Patients presenting in a non-thrombectomy-capable center >4.5 h from symptom onset, or with unknown time onset in a center without advanced imaging, are practically ineligible for ARTs. If CTA is readily available, LVO patients could be transferred to the nearest thrombectomy-capable center. In the case where CTA is unavailable, physicians may use established stroke scales for LVO-AIS prediction, as previously discussed.

Interestingly, non-randomized data from an international multicenter registry-based study (Thrombolysis in Stroke With Unknown Onset Based on Non-Contrast Computerized Tomography-TRUST/CT) and from a meta-analysis showed that IVT with alteplase in patients with unknown time onset based on non-contract CT (NCCT) is probably safe and effective [27,28]. The imaging exclusion criteria included an Alberta Stroke Program Early CT Score (ASPECTS) of <7, and clear hypodensity or involvement of >1/3 of the middle cerebral artery (MCA) territory. Nonetheless, two recent RCTs of IVT with tenecteplase in wake-up stroke (Tenecteplase in Wake-up Ischaemic Stroke Trial-TWIST) and in minor LVO-stroke <12 h from symptom onset (Tenecteplase versus standard of care for Minor Ischemic Stroke With Proven Occlusion-TEMPO 2) did not demonstrate better outcomes with tenecteplase, whereas in TEMPO-2 there were more deaths in the active treatment group [29,30]. Patients were excluded for hypoattenuation in >1/3 of the MCA territory (TWIST) or for a region of well-evolved infarction and/or ASPECTS < 7 (TEMPO-2). Ongoing trials, such as the Extending the Time window of IV thrombolysis by tenecteplase up to 2 h extension trial (EXIT-BT2), will investigate the feasibility of administrating tenecteplase up to 6 h from symptom onset based on NCCT [31]. Currently, IVT is not recommended in an extended or unknown time window without perfusion imaging or magnetic resonance imaging (MRI) [14].

### 2.4. Patients Presenting >4.5 h from Symptom Onset or with Unknown Time Onset in a Non-Thrombectomy-Capable Center but with Advanced Imaging (Figure 4)

**Figure 4:**
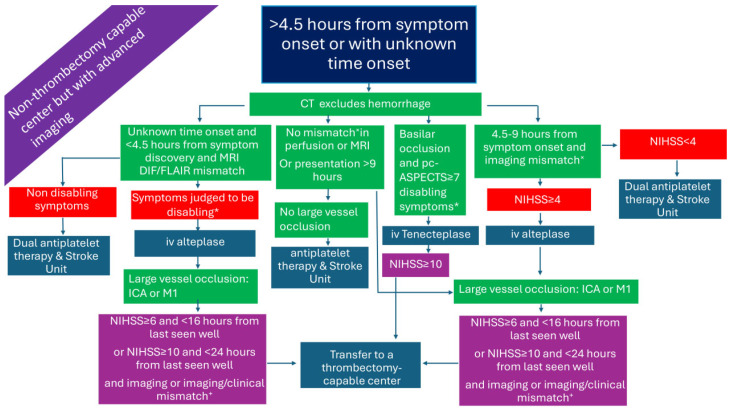
Management of AIS patients presenting >4.5 h from symptom onset in a non-thrombectomy-capable center but with advanced imaging. AIS: acute ischemic stroke; DIF/FLAIR: diffusion/Fluid-Attenuated Inversion Recovery; ICA: internal carotid artery; iv: intravenous; M1: M1 segment of the middle cerebral artery; M2: M2 segment of the middle cerebral artery; NIHSS: National Institutes of Health Stroke Scale; pc-ASPECTS: posterior circulation Alberta stroke program early CT score. * symptoms that interfere with daily living and previous activities of the patient. ^×^ Perfusion lesion–ischemic core mismatch ratio > 1.2, absolute difference in volume > 10 mL, and ischemic-core volume < 70 mL. ^+^ penumbral volume to infarct volume of ≥1.8, with an absolute penumbral volume of ≥15 mL and an estimated volume of the ischemic core < 70 mL (DEFUSE-3 [54]), or ≥80 years old and NIHSS ≥ 10 and infarct core < 21 mL, or <80 years old and NIHSS ≥ 10 and infarct core < 31 mL, or <80 years old and NIHSS ≥ 20 and infarct core 31 = 51 mL (DAWN [55]).

Both European and American guidelines endorse IVT treatment with alteplase based on diffusion-weighted imaging/fluid-attenuated inversion recovery (DWI/FLAIR) mismatch on brain MRI in patients with wake-up/unknown time stroke onset who will not undergo MT [14,32]. In addition, the European Stroke Organization (ESO) guidelines also recommend IVT with alteplase for wake-up patients or those in the extended time window up to 9 h from symptom onset based on the clinical and imaging criteria (Figure 4) of the Extending the Time for Thrombolysis in Emergency Neurological Deficits (EXTEND) trial [33].

However, the clinical dilemma arises when the patient is also eligible for EVT. For these cases, there is limited and conflicting data in the literature [34]. A study from Germany, on patients with unknown stroke onset undergoing EVT, found that the time preceding IVT was associated with higher sICH rates (14.6%, 95% CI, 3.3% to 25.6%, *p* < 0.01), after adjustment for ASPECTS and net water uptake CT [35]. In contrast, in a retrospective analysis of a French patient cohort with unknown time of stroke onset and DWI/FLAIR mismatch, bridging treatment was independently associated with better outcomes compared to only IVT [36]. A much larger Italian patient cohort study of individuals with unknown stroke onset or onset ≤ 6 h and an ASPECTS 10, showed that bridging therapy resulted in better functional and radiological outcomes [37].

The recent ESO guidelines suggest, as an expert opinion, administering IVT with alteplase in patients with unknown time onset and LVO, according to the Efficacy and Safety of MRI-Based Thrombolysis in Wake-Up Stroke trial (WAKE-UP) criteria, regardless of whether they present to a thrombectomy, or non-thrombectomy, center, before proceeding with EVT [14,38]. For those with LVO presenting between 4.5 h and 9 h and fulfilling the EXTEND criteria, IVT is suggested for patients presenting in a non-thrombectomy center followed by direct transfer for EVT [14].

Recently, four RCTs have used advanced acute imaging to extend IVT with tenecteplase beyond the conventional 4.5 h time window. The MRI-guided thrombolysis for stroke beyond time window by tenecteplase (ROSE-TNK) was a phase II study conducted in China that used similar imaging criteria to the WAKE-UP trial. Eligible patients were required to have a National Institutes of Health Stroke Scale (NIHSS) score > 5, stroke onset (or last seen well) between 4.5 and 24 h, and no planned EVT. Initial brain-CT scan was followed by brain-MRI to look for DWI/FLAIR mismatch. Other imaging inclusion criteria included DWI-detected acute infarct ≤ 1/3 of the MCA, ≤1/2 of the anterior cerebral artery (ACA), ≤1/2 of the posterior cerebral artery territory (PCA) territory, or <70 mL DWI infarct volume. Patients receiving tenecteplase were more likely to experience early neurological improvement, whereas no sICH occurred. Other clinical outcomes did not differ between groups [39].

The Thrombolysis in Imaging-Eligible, Late-Window Patients to Assess the Efficacy and Safety of Tenecteplase study (TIMELESS) randomized LVO stroke patients to either IVT with tenecteplase or placebo within 4.5 to 24 h from symptom onset, with the majority of them undergoing EVT. Perfusion imaging criteria were used for patient selection (mismatch ratio > 1.8, absolute difference in volume > 15 mL, and ischemic core volume < 70 mL). Although tenecteplase increased recanalization rates at 24 h and did not increase sICH rates, the study was negative with respect to the primary end point of the median modified Rankin Score (mRS) at 90 days [40].

The Tenecteplase Reperfusion Therapy in Acute Ischemic Cerebrovascular Events–III (TRACE-III) trial, investigating the administration of tenecteplase in LVO-AIS within 4.5 to 24 h from symptom onset, employed imaging selection criteria consistent with those used in the TIMELESS trial; however, patients were not planned to undergo additional EVT. The trial was positive in relation to the primary endpoint (excellent functional outcome [mRS 0–1] at 3 months) without any safety concerns [41]. The Chinese Acute Tissue-Based Imaging Selection for Lysis in Stroke—Tenecteplase trial (CHABLIS-T) demonstrated higher recanalization rates with tenecteplase compared to placebo in LVO-AIS patients selected with the aid of perfusion imaging and treated within 4.5 to 24 h from symptom onset. However, 3-month clinical outcomes were not modified by tenecteplase [42].

A recent meta-analysis of the ROSE-TNK, TWIST, and TIMELESS studies investigating IVT treatment with tenecteplase for AIS in the extended time window found that tenecteplase was associated with higher odds of 3-month excellent functional outcome compared to controls (RR  =  1.17; 95% CI , 1.01–1.36; I2  =  0%), whereas sICH (RR  =  1.67; 95% CI, 0.70–4.00; I2  =  0%) and 3-month mortality (RR  =  1.10; 95% CI ,  0.81–1.49; I2  =  0%) were similar [43].

The most recently published RCT on late time window IVT was conducted in China. The HOPE trial was positive for the primary endpoint of mRS 0–1 at 90 days (adjusted risk ratio 1.52 [95% CI, 1.14–2.02]; *p* = 0.004). Patients, not planned for EVT, received alteplase or placebo with stroke onset or the midpoint between the last known well time and symptom recognition between 4.5 and 24 h. Imaging eligibility was based on perfusion studies and was identical to that of the EXTEND trial: ischemic core volume ≤ 70 mL, hypoperfused tissue/ischemic core volume of at least 1.2, and penumbra of at least 10 mL Although not a perquisite, 98% of the patients had LVO or distal medium vessel occlusion (DMVO). Rates of sICH were higher in the alteplase group, but mortality rates were similar [44].

Taken together, these data suggest that, at present, tenecteplase may be considered only as an off-label (without official regulatory approval) and non-guideline-based treatment for up to 24 h after symptom onset in patients selected with advanced imaging. The same applies to IVT with alteplase beyond 9 h, with the aid of perfusing imaging. Importantly, replication of these results in Western patient cohorts is needed before the therapeutic window for IVT can be officially extended.

### 2.5. Patients Presenting >4.5 h from Symptom Onset or with Unknown Time Onset in a Thrombectomy-Capable Center and Without Advanced Imaging (Figure 5)

As previously mentioned, IVT is not currently recommended in patients presenting >4.5 h from symptom onset without advanced imaging. Patients with LVO presenting up to 6 h from symptom onset have a strong indication for EVT [32,45]. Based on a meta-analysis of early time window RCTs, EVT is suggested by the ESO/European Society for Minimally Invasive Neurological Therapy (ESMINT) guidelines, as an expert opinion, up to 7 h and 18 min from symptom onset without the need for advanced imaging [45,46]. In line with the Endovascular Treatment for Small Core and Anterior Circulation Proximal Occlusion (ESCAPE) trial imaging criteria, it is also suggested that EVT might be offered to patients presenting up to 12 h after symptom onset, provided they have at least ASPECTS of 6 and at least moderate collaterals on CTA [47].

Τhe prospect of further extending the time window for EVT has been investigated in several non-randomized studies. A single center study observed similar clinical outcomes between patients selected with CT-ASPECTS (>6 for the late time window) in the early versus late (6–24 h) time window [48]. In the same context, a multicenter study using data from the Patient selection Multicenter Clinical Registry of Endovascular treatment for Acute ischemic stroke in the Netherlands (MR CLEAN Registry) found similar outcomes between patients presenting in the early (<6.5 h) versus late (>6.5 h) time window, the latter selected on the basis of a favorable ASPECTS and collateral status [49]. A meta-analysis of six non-randomized studies comprising 3,384 patients demonstrated similar rates of functional independence (odds ratio [OR] 1.03, 95% CI, 0.87–1.22; *p* = 0.71) and sICH (OR 1.09, 95% CI, 0.58–2.04; *p* = 0.80) between LVO patients selected for EVT between 6 and 24 h based on NCCT or CTP compared to those treated within the standard time window of 0–6 h [50]. Patients imaged with CTP had higher rates of successful reperfusion (OR 1.31, 95% CI, 1.05–1.64; *p* = 0.015) and lower rates of mortality (OR 0.79, 95% CI, 0.65–0.96; *p* = 0.017).

The selection of patients for EVT, presenting within the 6–24 h time window, with NCCT versus CTP or MRI, was studied in a multinational cohort study that documented no significant differences between the two groups. Most patients in the NCCT were selected based on favorable ASPECTS ≥ 5 [51]. A very similar retrospective study analyzing data from the Stroke Thrombectomy and Aneurysm (STAR) collaboration also documented the safety and efficacy of NCCT-based (ASPECTS > 6) selection of patients for EVT in the late time window [52].

A pivotal RCT aiming to simplify inclusion criteria for EVT in anterior circulation and late presentation strokes was the Multicenter Randomized Clinical trial of Endovascular treatment of Acute Ischemic Stroke in the Netherlands for LATE arrivals (MR CLEAN-LATE) trial. The trial enrolled anterior circulation LVO patients between 6 and 24 h from symptom onset, provided they had at least some collaterals and a hypodensity of no more than a third of the MCA territory [53]. Of note, patients with similar clinical and imaging criteria with the previously published late time window EVT trials, Endovascular Therapy Following Imaging Evaluation for Ischemic Stroke 3 (DEFUSE-3) and DWI or CTP Assessment with Clinical Mismatch in the Triage of Wake Up and Late Presenting Strokes Undergoing Neurointervention With Trevo (DAWN), were excluded [54,55]. The trial was positive with regard to the primary endpoint, which was the median mRS score at 3 months (3 [IQR 2–5] vs. 4 [2,3,4,5,6]) with similar death rates between groups. Interestingly, 30% of patients had proximal M2 occlusions [53].

In conclusion, EVT might be offered up to 12 h after symptom onset, without the need for advanced imaging, provided there is a favorable ASPECTS and collateral grade according to the ESCAPE trial. Extending the time window for EVT up to 24 h with the aid of NCCT and CTA should currently be considered as a non-guideline-based treatment option.

### 2.6. Patients Presenting >4.5 h from Symptom Onset or with Unknown Time Onset in a Thrombectomy-Capable Center and with Advanced Imaging (Figure 6)

Non-LVO patients, otherwise fulfilling the WAKE-up and EXTEND criteria, may receive IVT according to the guidelines [14,32]. For LVO-AIS, the ESO guidelines suggest bridging IVT with EVT for wake-up/unknown onset and direct EVT for patients presenting within the 4.5–9 h window and fulfilling, in addition, the EXTEND criteria [14]. EVT in late time windows could be offered up to 16 or 24 h according to the DEFUSE-3 and DAWN trials criteria, respectively [54,55].

## 3. Large Core Ischemic Stroke

A large ischemic core is defined as an ASPECTS of less than 6 on NCCT or DWI, or as a severely hypoperfused area—typically with a relative cerebral blood flow (rCBF) of <30% on CTP or a volumetric estimation of the DWI lesion exceeding 50 mL or 70 mL [56,57]. Patients with large core AIS were excluded for the initial EVT trials, both in the early and late time windows. However, following the publication of five positive RCTs (Recovery by Endovascular Salvage for Cerebral Ultra-Acute Embolism–Japan Large Ischemic Core Trial [RESCUE-Japan LIMIT], Study of Endovascular Therapy in Acute Anterior Circulation Large Vessel Occlusive Patients with a Large Infarct Core [ANGEL-ASPECT], A Randomized Controlled Trial to Optimize Patient’s Selection for Endovascular Treatment in Acute Ischemic Stroke [SELECT2], Efficacy and Safety of Thrombectomy in Stroke With Extended Lesion and Extended Time Window [TENSION], and Large Stroke Therapy Evaluation [LASTE]), EVT indication is being extended even to this group of LVO patients [58,59,60,61,62]. A sixth RCT (Thrombectomy for Emergent Salvage of Large Anterior Circulation Ischemic Stroke [TESLA]) was neutral regarding the primary endpoint of utility-weighted mRS, but with a trend towards improved outcomes in favor of the EVT group [63]. Two RCTs (RESCUE-Japan LIMIT and LASTE) primarily used MRI to estimate the ASPECTS, and another two (ANGEL-ASPECT and SELECT2) allowed for the inclusion of patients with <3 ASPECTS or >6 ASPECTS provided they had a CTP-core volume between 70 and 100 mL or >50 mL, respectively. Regarding time from symptom onset, two studies recruited patients within 6 h (also allowed FLAIR negative up to 24 h from last known to be well), three studies up to 24 h, and one up to 11 h.

A study-level meta-analysis and meta-regression of the six RCTs found that EVT was associated with an improved mRS score (generalized OR, 1.6, 95% CI, 1.4–1.8), and independent ambulation (RR 1.9; 95% CI, 1.5–2.5), as well as reduced mortality (RR 0.9; 95% CI, 0.8–1.0) at 90 days. The results were mainly driven by the more pronounced benefits observed in the ASPECTS 3–5 group [64]. Nevertheless, EVT for large core AIS is not currently endorsed by the latest guidelines. The strongest evidence comes from cases with ASPECTS 3–5, which may be prioritized for treatment. When patients present in centers with readily available advanced imaging, CTP and/or DWI-MRI can further aid in a more accurate estimation of the extent and location of irreversible lesions. Things become more complicated with respect to the time from symptom onset, but even treatment of patients up to 24 h may be considered.

## 4. Distal Medium Vessel Occlusions (DMVO)

Patients with distal occlusions were severely under-represented in the pivotal EVT-RCTs [65]. Subsequently, three RCTs on EVT for DMVO were presented at the most recent international stroke conference in Los Angeles, USA (2025), and two of them have already been published (Endovascular therapy plus best medical treatment (BMT) versus BMT alone for medium distal vessel occlusion stroke [DISTAL], and Endovascular Treatment to Improve outcomes for Medium Vessel Occlusions [ESCAPE-MeVO]. All three studies (DISTAL, ESCAPE-MeVO and Evaluation of Mechanical Thrombectomy in Acute Ischemic Stroke Related to a Distal Arterial Occlusion [DISCOUNT]) did not meet their primary endpoint, which was defined as either a difference in the distribution of mRS scores at 90 days or the proportion of patients achieving mRS score of 0–1 or 0–2 at 90 days. The DISTAL trial enrolled patients with occlusions in co-dominant or non-dominant M2–M4, A1–A3, and P1–P3 segments; the ESCAPE-MeVO trial included patients with M2–M3, A2–A3, and P2–P3 occlusions; and the DISCOUNT trial investigated patients with distal M2–M3, A1–A3, and P1–P3 occlusions [66,67,68].

A study-level meta-analysis with random effects modeling did not demonstrate a benefit of EVT over best medical treatment (BMT) with respect to functional outcome (RR 0.92, 95% CI, 0.80–1.06, *p* = 0.272). Mortality rates were similar; however, rates of sICH were doubled in the EVT group (OR 2.38, 95% CI, 1.35–4.20, *p* = 0.003) [65]. Finally, in patients with DMVO, a recent meta-analysis combining randomized and observational data has shown that EVT may be associated with higher symptomatic intracranial hemorrhage rates without improving functional outcomes [69]. Therefore, EVT for DMVO has not yet proved its efficacy and should not be offered as a standard therapeutic option. By contrast, EVT for dominant M2-MCA occlusions may yield outcomes comparable to those for M1-MCA occlusions and could be considered for treatment, especially when patients present with high NIHSS scores and are not eligible for IVT [70,71].

## 5. Basilar Artery Occlusion

Since basilar artery occlusion (BAO) stroke is relatively rare, there are no RCTs specifically comparing IVT versus no acute recanalization treatment in this population. The recently published Extending the Time Window for Thrombolysis in Posterior Circulation Stroke Without Early CT Signs (EXPERTS) study randomized patients with mainly mild posterior circulation strokes, not necessarily harboring BAO, and with CT-evaluated ASPECTS > 6, to receive IVT with alteplase or placebo. IVT was beneficial with a significant increase in functional independence at 90 days [72]. Obviously, BAO patients are treated with IVT similarly to other AIS cases. Concerning the time frame and imaging criteria, non-randomized observational data have shown that IVT may be safe and effective for patients presenting without extensive posterior circulation ischemic changes (typically a posterior ASPECTS [pc-ASPECTS] score of ≥6) and at time points up to 48 h from symptom onset [73,74,75]. In a subsequent multicenter cohort study, IVT alone was superior to EVT or EVT + IVT, although this benefit was offset in cases presenting >6 h (>30% of patients in the IVT only group). Median pc-ASPECTS was 8 with 12% of the cases having pc-ASPECTS < 8 [76].

A recent meta-analysis of non-RCTs of IVT-treated BAO strokes in the extended time window documented rates of favorable functional outcome (FFO) at 90 days of 34% (95% CI, 17–56), with an average onset to treatment time of 328.9 min (95% CI: 249.1–434.3) and sICH rates of 9% (95% CI: 5–15) [77]. In addition, EVT alone compared to bridging therapy was associated with reduced odds of functional independence (29% vs. 38%; RR 0.78, 95% CI, 0.68–0.88, *p* < 0.001), and independent ambulation (39% vs. 45%; RR 0.89, 95% CI, 0.82–0.98, *p* = 0.01), but higher mortality rates (36% vs. 28%, RR 1.22, 95% CI, 1.08–1.37, *p* = 0.001) in a meta-analysis of 2 RCTs and 10 cohort studies [78]. Accordingly, the joint ESO and ESMINT (European society for minimally invasive neurological therapy) guidelines suggest IVT for BAO up to 24 h after symptom onset for cases without extensive bilateral and/or brainstem ischemic changes on CT or MRI [79]. In Table 1, we present guideline-based IVT treatment recommendations versus off-label IVT treatment considerations, as well as non-guideline-based EVT treatment considerations.

Four RCTs have evaluated the efficacy and safety of EVT for basilar or vertebrobasilar artery occlusion. The Acute Basilar Artery Occlusion: Endovascular Interventions vs. Standard Medical Treatment (BEST) trial recruited patients up to 8 h from symptom onset based on a favorable pc-ASPECTS (median 8). IVT was administered to 1/3 of the participants. Although the trial was negative regarding the primary outcome of mRS ≤ 3, the result might have been influenced by the effect of the crossovers [80]. The Basilar Artery International Cooperation Study (BASICS) which recruited patients with BAO within 6 h of symptom onset and without extensive bilateral brainstem infarction on CT was negative for the primary outcome of the favorable functional outcome (defined as mRS scores 0–3). Interestingly, almost 80% of patients received IVT within a median of about 2 h after symptom onset, contributing to a relatively high FFO and recanalization rates of 38% and 56% in the controls, respectively [81].

Subsequently, two Chinese RCTs published in 2022 were positive. The Basilar Artery Occlusion Chinese Endovascular Trial (BAOCHE) included patients presenting between 6 and 24 h after symptom onset with a pc-ASPECTS of ≥6 and without large brain stem infarct, defined as a Pons-Midbrain Index of ≥2 points, while less than 20% of patients received IVT [82,83]. The trial was positive regarding the primary endpoint (mRS scores 0–3 at 90 d; adjusted rate ratio, 1.81; 95% CI, 1.26–2.60; *p* < 0.001) [83]. Similarly, the Endovascular Treatment for Acute Basilar Artery Occlusion (ATTENTION), which enrolled BAO patients within 12 h of stroke onset with a pc-ASPECTS of ≥6 for those <80 years old and ≥8 for those ≥80 years old, and with less than 33% receiving IVT, was positive for the primary endpoint (mRS 0–3 at 90 d; adjusted rate ratio, 2.06; 95% CI, 1.46–2.91, *p* < 0.001) [84]. Interestingly, the authors conducted an individual patient data meta-analysis from the ATTENTION and ATTENTION IA trials (randomized patients with posterior circulation stroke [>70% BAO] up to 24 h from symptom onset, ps-ASPECTS ≥ 6, and successful EVT to intra-arterial tenecteplase, or not) and failed to find significant differences in the 3-month mRS scores of 0–3 between patients selected with CTP and NCCT [85,86].

The ESO guidelines recommend EVT plus BMT for BAO patients who present within 24 h of symptom onset and without extensive ischemic changes (ps-ASPECTS ≥ 7), provided they have an NIHSS score ≥ 10. The authors also noted that the effect of EVT may be diminished in patients who receive IVT within 6 h of symptom onset. Perfusion imaging is not considered essential for patient selection [79].

Of note, posterior circulation stroke is a rather heterogeneous entity, given the high variability in vascular anatomy, which also results in variability in clinical presentation and outcomes. Such disparities may be addressed through the development of posterior circulation-specific stroke scales—since the NIHSS may underestimate the severity of neurological deficits—, the more extensive use of CT/MR angiography in patients presenting with symptoms is indicative of posterior circulation stroke, and of the potential categorization of patients based on the severity of clinical presentation and the specific artery involved.

## 6. Patients with Minor Stroke and LVO

Approximately 10% of patients with LVO present with minor (NIHSS < 6) strokes [87]. These patients were excluded from the RCTs. However, early neurological deterioration (END; increase in NIHSS ≥ 4) is far more common in LVO-related AIS, occurring in 10–20% of patients treated medically (with or without IVT) and associated with worse outcomes [88,89,90]. More proximal occlusion sites and longer thrombus lengths, as well as markers of the severity of hypoperfusion in perfusion imaging, are associated with END [90,91]. Still, rescue EVT immediately after END might improve outcomes [89].

The ESO/ESMINT guidelines suggest considering EVT for LVO patients with low NIHSS presenting with disabling symptoms (such as motor deficit, aphasia, and hemianopia) and in cases with END [45]. In a recent meta-analysis of 11 observational studies, EVT was not associated with reduced disability at 90 days (common odds ratio, 0.92 [95% CI, 0.60–1.41]) but with increased risk of sICH (risk ratio, 3.53 [95% CI, 2.35–5.31]). Approximately one half of EVT-treated patients received additional IVT versus 81% in the BMT group [19]. Therefore, careful selection for EVT eligibility based on the estimated risk of END is important when evaluating patients with LVO and low NIHSS scores.

## 7. Discussion

In Table 2, we present an overview of the RCTs discussed in the manuscript.

**Figure 1:**
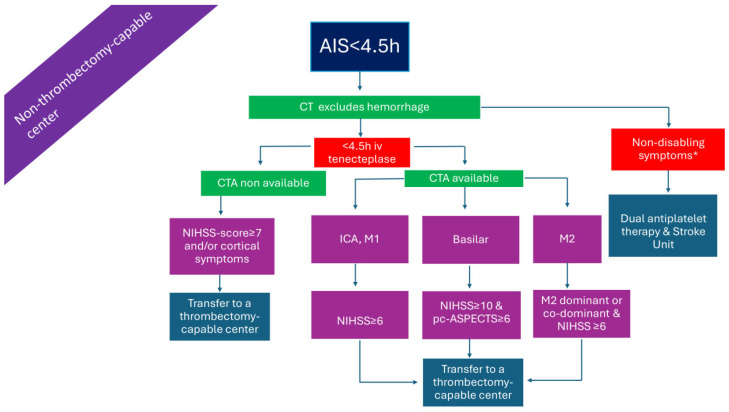
Management of AIS patients presenting within 4.5 h from symptom onset in a non-thrombectomy-capable center. AIS: acute ischemic stroke; CTA: computed tomography angiography; ICA: internal carotid artery; iv: intravenous; M1: M1 segment of the middle cerebral artery; M2: M2 segment of the middle cerebral artery; NIHSS: National Institutes of Health Stroke Scale; pc-ASPECTS: posterior circulation Alberta stroke program early CT score. * symptoms that interfere with daily living and previous activities of the patient.

**Figure 2:**
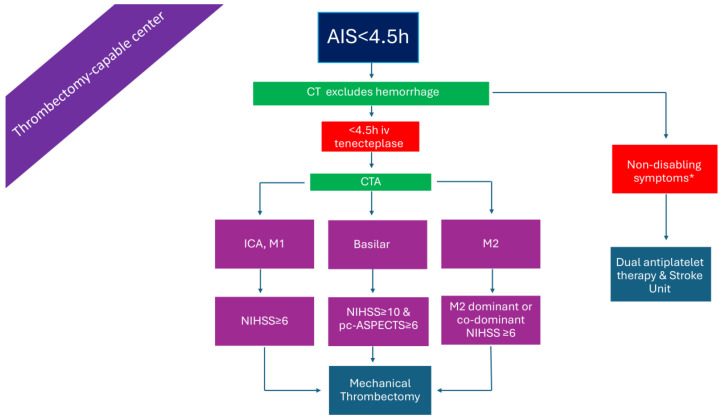
Management of AIS patients presenting within 4.5 h from symptom onset in a thrombectomy-capable center. AIS: acute ischemic stroke; CTA: computed tomography angiography; ICA: internal carotid artery; iv: intravenous; M1: M1 segment of the middle cerebral artery; M2: M2 segment of the middle cerebral artery; NIHSS: National Institutes of Health Stroke Scale; pc-ASPECTS: posterior circulation Alberta stroke program early CT score. * symptoms that interfere with daily living and previous activities of the patient.

**Figure 3:**
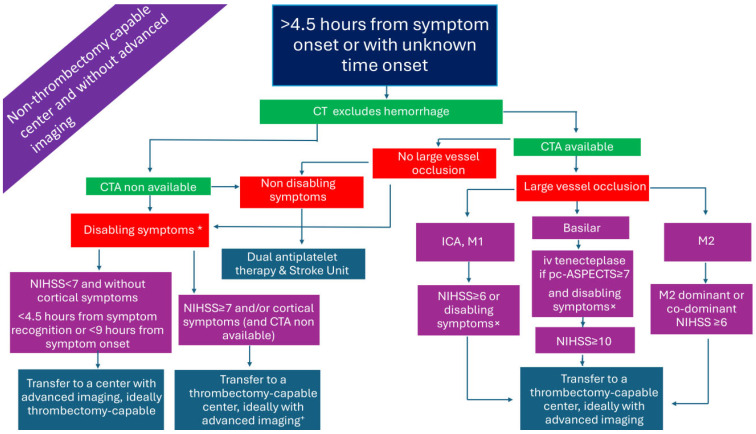
Management of AIS patients presenting >4.5 h from symptom onset in a non-thrombectomy-capable center and without advanced imaging. AIS: acute ischemic stroke; CTA: computed tomography angiography; ICA: internal carotid artery; M1: M1 segment of the middle cerebral artery; M2: M2 segment of the middle cerebral artery; NIHSS: National Institutes of Health Stroke Scale; pc-ASPECTS: posterior circulation Alberta stroke program early CT score. * symptoms that interfere with daily living and previous activities of the patient. ^×^ significant motor deficit or aphasia or hemianopia. ^+^ especially if symptom onset >6 h or when mechanical thrombectomy cannot be initiated within 7 h and 18 min from symptom onset.

**Figure 5:**
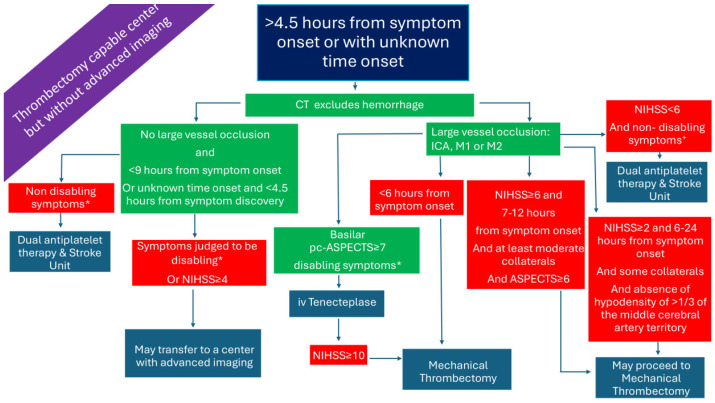
Management of AIS patients presenting >4.5 h from symptom onset in a thrombectomy-capable center without advanced imaging. AIS: acute ischemic stroke; ICA: internal carotid artery; iv: intravenous; M1: M1 segment of the middle cerebral artery; M2: M2 segment of the middle cerebral artery; NIHSS: National Institutes of Health Stroke Scale; pc-ASPECTS: posterior circulation Alberta stroke program early CT score. ^+^ e.g., significant motor deficit or aphasia or hemianopia. * symptoms that interfere with daily living and previous activities of the patient.

**Figure 6:**
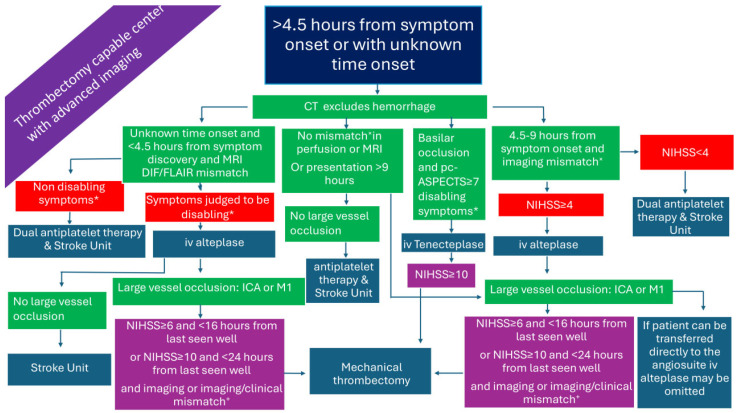
Management of AIS patients presenting >4.5 h from symptom onset in a thrombectomy-capable center with advanced imaging. AIS: acute ischemic stroke; ICA: internal carotid artery; iv: intravenous; M1: M1 segment of the middle cerebral artery; M2: M2 segment of the middle cerebral artery; NIHSS: National Institutes of Health Stroke Scale; pc-ASPECTS: posterior circulation Alberta stroke program early CT score. * symptoms that interfere with daily living and previous activities of the patient. ^×^ Perfusion lesion–ischemic core mismatch ratio > 1.2, absolute difference in volume > 10 mL, and ischemic-core volume < 70 mL. ^+^ penumbral volume to infarct volume of ≥1.8, with an absolute penumbral volume of ≥15 mL and an estimated volume of the ischemic core < 70 mL (DEFUSE-3 [54]), or ≥80 years old and NIHSS ≥ 10 and infarct core < 21 mL, or <80 years old and NIHSS ≥ 10 and infarct core < 31 mL, or <80 years old and NIHSS ≥ 20 and infarct core 31 = 51 mL (DAWN [55]).

**Figure 7:**
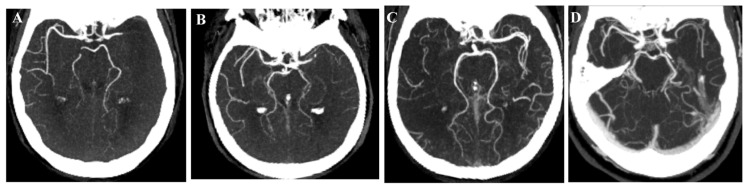
Collateral grading: (**A**): grade 0 (absence of collaterals). (**B**): grade 1 (collateral filling ≤ 50%, but >0%). (**C**): grade 2 (collateral filling > 50%, but <100%). (**D**): grade 3 (100% collateral filling) scored in comparison to the entire contralateral middle cerebral artery territory [86].

**Figure 8:**
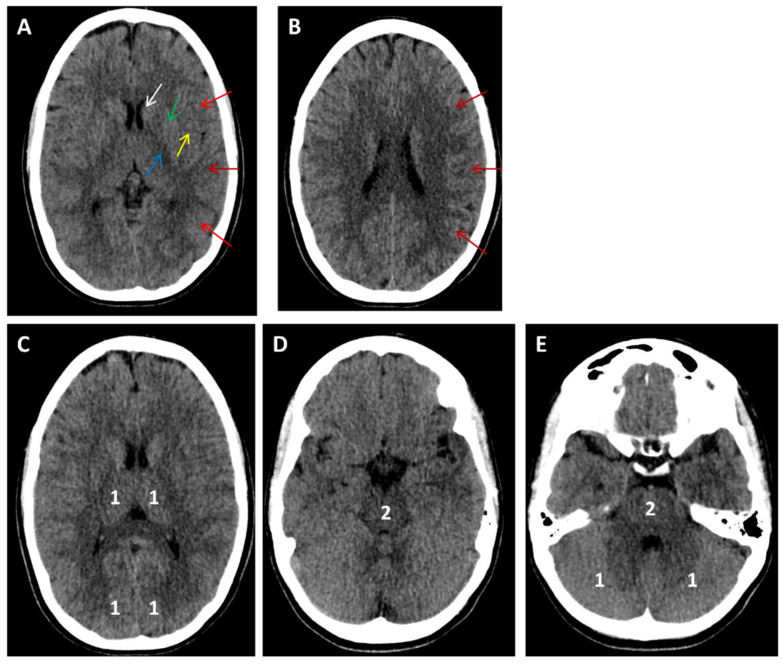
Alberta stroke program early CT score (ASPECTS; shown in panels (**A**,**B**)) and posterior circulation ASPECTS (pc-ASPECTS; shown in panels (**C**–**E**). (**A**): Regions assessed for early ischemic changes in ASPECTS at the ganglionic level shown with arrows (white: caudate; green: lentiform nucleus; yellow: insula; blue: internal capsule; red arrows: M1, M2, and M3 MCA cortex). Regions assessed for early ischemic changes in ASPECTS at the supraganglionic level shown with red arrows (M4, M5, and M6 MCA cortex). One point is subtracted for each region. (**C**–**E**): Regions assessed for early ischemic changes in the pc-ASPECTS. Numbers indicate the points subtracted for each region.

In Figure 1, Figure 2, Figure 3, Figure 4, Figure 5 and Figure 6, we present a recommended treatment workflow based on imaging modality and EVT availability. Figure 7 shows examples of different collateral grades on single-phase brain CTA [92]. Figure 8 demonstrates the regions assessed for early ischemic changes in the ASPECTS and pc-ASPECTS.

One of the major priorities in stroke medicine involves the expansion of ARTs to encompass a larger proportion of AIS patients. Two of the most promising and broadly applicable strategies in this context are the extension of the therapeutic time window and the simplification of imaging requirements.

Ongoing RCTs are aiming to address these therapeutic needs. The EXIT-BT2 study (NCT06010628) extends the therapeutic window of IVT with tenecteplase by 2 h based solely on NCCT [31]. The “Extending the Time Window for Intravenous Tenecteplase in Patients With Distal Medium Vessel Occlusions Stroke” (TNK-MeVO [NCT06559436]) is recruiting Chinese patients with DMVO to administer tenecteplase or placebo within 4.5–24 h from symptom onset. Imaging eligibility includes <50% ischemic core in the affected territory at least 6 h after symptom onset, as depicted on DWI-MRI, CTP, or NCCT (hypodensity and loss of gray-white border), allowing enrollment of patients without the use of advanced imaging. Eligible for recruitment in the “Randomization to Extend Stroke Intravenous ThromboLysis In Evolving Non-Large Vessel Occlusion With TNK” (RESILIENT [EXTEND-IV]) (NCT05199662) trial are patients with clinical or imaging mismatch, including those with NIHSS score ≥ 4 and NCCT with <50% involvement of the vascular territory corresponding to the clinical manifestation.

On the other hand, the Chinese trial “Intravenous Thrombolysis With rhTNK-tPA for Acute Non-large Vessel Occlusion in Extended Time Window” (OPTION trial [NCT05752916]) requires the presence of a target mismatch on CTP in order to enroll patients without LVO to receive tenecteplase or placebo within 4.5–24 h from symptom onset. Perfusion imaging was also required in the Australian “Extending the time window for Tenecteplase by Effective Reperfusion of peNumbrAL tissue in patients with Large Vessel Occlusion” (ETERNAL-LVO) trial (NCT04454788) for the selection of LVO-AIS up-to 24 h from symptom onset to receive tenecteplase, alteplase, or placebo before undergoing EVT [93]. This study was terminated due to loss of equipoise in the 0–4.5 h window, which enrolled the majority of patients, after the publication of other RCTs comparing tenecteplase with alteplase. In addition, the BI 1123-0060TENACITY, a Phase III, prospective, randomized, open-label, blinded endpoint assessment (PROBE) trial, will assess the efficacy and safety of intravenous tenecteplase versus standard of care in patients with acute ischemic stroke (including wake-up stroke), last known well > 4.5 h, with imaging evidence of salvageable ischemic tissue (Table 1).

The use of advanced imaging is not mandatory in the “Extending the Time Window for Tenecteplase by Recanalization of Basilar Artery Occlusion in Posterior Circulation Stroke” (POST-ETERNAL) trial (NCT05105633), which is recruiting AIS patients with BAO presenting within 24 h with pcASPECTS ≥ 7. Similar time-window and imaging criteria are applied in the “Efficacy and Safety of Tenecteplase Intravenous Thrombolysis in Acute Posterior Circulation Ischemic Stroke Within 4.5–24 h After Onset” (NCT07094763) trial, which enrolls patients with posterior circulation AIS (Table 3).

With respect to EVT, ongoing RCTs from China will enroll patients beyond the 24 h time window, either without the use of advanced imaging, like the “Thrombectomy Versus Best Medical Management in Large Vessel Occlusion Stroke Patients Presenting Beyond 24 Hours and With Presence of Collateral Flow on CT Angiography” (NCT06654375) and the “Basilar Artery Occlusion Chinese Endovascular Trial in the Extended Time Window” (BAOCHE2, NCT06560203), or with the use of advanced imaging (perfusion or MRI) like the “Large Artery Occlusion Treated in Extended Time with Mechanical Thrombectomy Trial” (LATE-MT, NCT05326932). Finally, based on the positive results of a retrospective observational international multicenter cohort study, the SELECT-LATE global RCT is planning to randomize, between 24 and 72 h from symptom onset, LVO-AIS patients with salvageable penumbra to receive EVT or the best medical therapy (Table 4) [94].

## 8. Conclusions

IVT and EVT are two powerful interventions in the arsenal of stroke medicine that have altered the natural course of AIS. However, their application is limited by the narrow therapeutic time window and requirement for advanced imaging when attempting to extend it. In the present manuscript, we present individualized treatment options according to different time windows of presentation, imaging modalities, and EVT availability, which may be followed in line with current international recommendations. We also present data from recent trials aiming to expand the use of ARTs in cases of delayed presentation without the use of advanced imaging, posterior circulation AIS, large infarcts, DMVOs, and LVO with low NIHSS. With ongoing research and technological advances, novel thrombolytics, such as tenecteplase, along with new microcatheters and interventional techniques, including intra-arterial thrombolysis post EVT, may enable broader implementation of ARTs in a safer and more effective manner. Moreover, the adoption of tenecteplase as the thrombolytic of choice, along with the simplification of imaging protocols and the minimization of imaging prerequisites for the implementation of ARTs, may prove valuable for low-income countries and resource-limited settings, allowing for faster, safer, and more cost-effective treatment of larger numbers of AIS patients.

Future RCTs are needed to address specific research gaps and to include under-represented populations, such as patients with multiple comorbidities, cancer, prior non-vitamin K antagonist anticoagulant (NOAC) therapy, renal failure, and the oldest old. Finally, most stroke RCTs assess effectiveness primarily through functional outcomes, typically measured by the mRS. However, patient-centered outcomes—such as quality of life or return to work—represent important aspects of everyday living that are not fully captured by the mRS and are increasingly emphasized in stroke research.

## Figures and Tables

**Table 1:** Guideline-based IVT treatment recommendations versus off-label IVT treatment considerations, as well as non-guideline-based EVT treatment considerations.

Off-Label, Guideline-Based IVT Treatment Considerations	Off-Label, Non-Guideline-Based IVT Treatment Considerations	Non-Guideline-Based EVT Treatment Considerations
IVT with alteplase in wake-up/unknown time stroke onset presenting within 4.5 h after symptom recognition, with DWI/FLAIR MRI mismatch (ESO, AHA/ASA guidelines)	IVT with tenecteplase ≤ 24 h from symptom onset/last seen well and presence of CTP/MRP mismatch	EVT ≤ 24 h from symptom onset/last seen well based on NCCT (ASPECTS ≥ 6)
IVT with alteplase ≤ 9 h from stroke onset and presence of CTP/MRP mismatch (ESO guidelines)		EVT for large core (ASPECTS ≤ 5) AIS ≤ 24 h from symptom onset/last seen well
Bridging therapy (IVT with alteplase plus EVT) in wake-up/unknown time stroke onset presenting within 4.5 h after symptom recognition, with DWI/FLAIR MRI mismatch and LVO (ESO guidelines)		EVT > 24 h from symptom onset/last seen well, with salvageable brain tissue
If presenting in a non-thrombectomy center, IVT with alteplase ≤ 9 h from stroke onset in the presence of CTP/MRP mismatch and LVO, as well as transfer to a thrombectomy-capable center (ESO guidelines)		
IVT for BAO ≤ 24 h without extensive bilateral and/or brainstem ischemic changes (pc-ASPECTS ≥ 7) on CT or MRI (ESO/ESMINT guidelines)		

AIS: Acute ischemic stroke; ASPECTS: Alberta Stroke Program Early CT Score; AHA/ASA: American Heart Association/American Stroke Association; BAO: basilar artery occlusion; DWI: diffusion weighted imaging; CTP: CT perfusion; ESMINT: European Society for Minimally Invasive Neurological Therapy; ESO: European Stroke Organization; EVT: endovascular treatment; FLAIR: fluid-attenuated inversion recovery; IVT: intravenous thrombolysis; LVO: large vessel occlusion; MRP: MR perfusion; NCCT: non-contrast CT; pc-ASPECTS: posterior circulation ASPECTS.

**Table 2:** Overview of the Randomized control trials discussed in the manuscript.

RCT	Intervention	Inclusion Criteria	Key Exclusion Criteria	Primary Outcome	Key Secondary Outcomes
BRIDGE-TNK [26]	iv TNK plus EVT vs. EVT	LVO: ICA, M1/M2-MCA, VB AIS ≤ 4.5 h	Non eligible for IVT	mRS 0–2 (90 d): TNK/EVT 52.9% vs. EVT 44.1% (unadjusted risk ratio, 1.20; 95% confidence interval, 1.01–1.43; *p* = 0.04)	successful reperfusion before EVT: TNK/EVT 6.1% vs. EVT 1.1% (adjusted risk ratio, 5.19; 95% CI, 1.51–17.84) sICH: TNK/EVT 8.5% vs. EVT 6.7% (adjusted risk ratio, 1.35; 95% CI, 0.74–2.44; *p* = 0.33) mortality (90 d) TNK/EVT 22.3% vs. EVT 19.9% (adjusted hazard ratio, 1.17; 95% CI, 0.81–1.69; *p* = 0.39)
TWIST [29]	TNK vs. no IVT based on NCCT	Wake up stroke NIHSS ≥ 3 Treatment within 4.5 h	Infarction > 1/3 of the MCA territory NCCT	mRS (90 d): adjusted OR 1.18, 95% CI 0.88–1.58; *p* = 0.27	Mortality (90 d): adjusted HR 1.29, 95% CI 0.74–2.26; *p* = 0.37 sICH: adjusted OR 2.17, 95% CI 0.53–8.87; *p* = 0.28
TEMPO 2 [30]	TNK vs. no IVT based on Multiphase CTA or CTP	last-seen-well ≤ 12 h TIA or minor stroke (NIHSS ≤ 5) Any acute intracranial occlusion (CTA or MRA) OR focal perfusion abnormality (CTP or MRP)	ASPECTS < 7 Planned EVT	return to baseline functioning on pre-morbid mRS (90 d): TNK 72% vs. control 75% (risk ratio [RR] 0.96, 95% CI 0.88–1.04, *p* = 0.29)	sICH: TNK 2% vs. control < 1% (RR 4.2; 95% CI 0.9–19.7, *p* = 0.059)
EXTEND [33]	ALT vs. no IVT based on perfusion imaging	Treatment within ≥4.5–9 h NIHSS ≥ 4 Penumbral mismatch: “hypo-perfusion to core” volume ratio ≥ 1.2 and absolute difference ≥ 10 mL (using a MR or CT Tmax > 6 s delay) between perfusion lesion and MR-DWI or CT-CBF core lesion ischaemic core lesion volume ≤ 70 mL on MR-DWI or CT-CBF	Infarct core > 1/3 MCA territory Planned EVT	mRS 0–1 (90 d): ALT 35.4% vs. placebo 29.5% (adjusted risk ratio, 1.44; 95% CI, 1.01 to 2.06; *p* = 0.04)	sICH ALT 6.2% vs. placebo 0.9% (adjusted risk ratio, 7.22; 95% CI, 0.97 to 53.5; *p* = 0.05)
WAKE-UP [38]	ALT vs. no IVT based on MRI	Wake-up stroke or stroke with unknown onset Treatment within 4.5 h DWI/FLAIR mismatch Age 18–80	Lesion > 1/3 MCA territory Planned EVT	mRS 0–1 (90 d): ALT 53.3% placebo 41.8% (adjusted odds ratio, 1.61; 95% CI, 1.09 to 2.36; *p* = 0.02)	Mortality (90): ALT 4.1% in the alteplase vs. placebo 1.2% (odds ratio, 3.38; 95% CI, 0.92 to 12.52; *p* = 0.07) sICH: ALT 2.0% vs. placebo 0.4% (odds ratio, 4.95; 95% CI, 0.57 to 42.87; *p* = 0.15)
ROSE-TNK [39]	TNK vs. no IVT based on MRI	NIHSS 6–25 Treatment 4.5–24 h DWI/FLAIR mismatch Age 18–80	DWI infarct > 1/3 of MCA territory or >1/2 of ACA territory or >1/2 of PCA DWI infarct volume > 70 mL Planned EVT	mRS 0–1 (90 d): TNK 52.5% vs. control 50.0% (unadjusted odds ratio, 1.11; 95% confidence interval 0.46–2.66; *p* = 0.82)	early neurological improvement: TNK vs. control 11 vs. 3, *p* = 0.03 no cases of sICH
TIMELESS [40]	TNK vs. no IVT based on CTP	Symptom onset within 4.5 to 24 h LVO: ICA, M1/M2-MCA ischemic core volume < 70 mL, mismatch ratio ≥ 1.8 and mismatch volume ≥ 15 mL	Non eligible for IVT	ordinal score on the mRS (90 d): adjusted common odds ratio for TNK vs. placebo 1.13 (95% CI, 0.82–1.57; *p* = 0.45)	mortality (90 d): TNK 19.7% vs. placebo 18.2% sICH: TNK 3.2% vs. placebo 2.3%
TRACE-III [41]	TNK vs. no IVT based on CTP	Symptom onset within 4.5 to 24 h LVO: ICA, M1/M2-MCA ischemic core volume < 70 mL, mismatch ratio ≥ 1.8 and mismatch volume ≥ 15 mL no access to EVT NIHSS 6–25	Hypodensity in >1/3 MCA territory on NCCT non eligible for IVT	mRS 0–1 (90 d): TNK 33.0% vs. no IVT 24.2% (relative rate, 1.37; 95% CI, 1.04–1.81; *p* = 0.03)	Mortality (90 d): TNK 13.3% vs. no IVT 13.1% sICH: TNK 3.0% vs. no IVT 0.8%
CHABLIS-T [42]	TNK vs. no IVT based on CTP	Symptom onset within 4.5 to 24 h LVO: ICA, M1/M2-MCA, ACA ischemic core volume < 70 mL, mismatch ratio ≥ 1.2 and mismatch volume ≥ 10 mL NIHSS ≥ 6 Age 18–80	Hypodensity in > 1/3 MCA territory non eligible for IVT	restoration of blood flow of >50% of the involved ischemic territory: TNK 33.3% vs. no IVT 10.8% (adjusted relative risk, 3.0; 95% CI, 1.6–5.7; *p* = 0.001)	recanalization rate: TNK 35.8% vs. no IVT 14.3%, adjusted relative risk, 2.5; 95% CI, 1.4–4.4; *p* = 0.002) no significant differences in clinical efficacy outcomes or sICH
HOPE [44]	ALT vs. no IVT based on CTP	Symptom onset within 4.5 to 24 h ischemic core volume < 70 mL, mismatch ratio ≥ 1.2 and mismatch volume ≥ 10 mL NIHSS 4–26	Planned EVT non eligible for IVT	mRS 0–1 (90 d): ALT 40% vs. control 26% (adjusted risk ratio, 1.52; 95%CI, 1.14–2.02; *p* = 0.004)	sICH: ALT 3.8% vs. control 0.51% (adjusted risk ratio, 7.34; 95%CI, 1.54–34.84; *p* = 0.01) mortality: ALT 11% vs. control 11% (adjusted risk ratio, 0.91; 95%CI, 0.52–1.62; *p* = 0.76)
ESCAPE [47]	EVT vs. no EVT	last-seen-well to randomization time < 12 h NIHSS > 5 LVO: ICA, M1/M2-MCA	no or minimal collaterals in a region greater than 50% of the MCA territory when compared to pial filling on the contralateral side low CBV and very low CBF ASPECTS < 6 on CTP region of low CBV and very low CBF > 1/3 on CTP	mRS shift analysis (90 d): common odds ratio EVT vs. no EVT, 2.6; 95% CI, 1.7–3.8; *p* < 0.001	mRS 0–2 (90 d): EVT vs. no EVT 53.0%, vs. 29.3% group; *p* < 0.001 sICH: EVT 3.7% vs. No EVT 2.7% (*p* = 0.75)
MR CLEAN-LATE [53]	EVT vs. no EVT based on CTA	6–24 h from symptom onset or last seen well LVO: ICA, M1/M2-MCA NIHSS > 2 presence of collateral flow in the MCA territory (grade 1, 2, or 3, see Figure 7)	Hypodensity in > 1/3 MCA territory	Median mRS (90 d): EVT 3 (IQR 2–5) vs. control 4 (2–6), (adjusted common OR 1.67, 95% CI, 1.20–2.32)	Mortality (90 d): EVT 24%] vs. control 30% (adjusted OR 0.72, 95% CI, 0.44–1.18) sICH: EVT 7% vs. control 2% (adjusted OR 4.59, 95% CI 1.49–14.10)
DEFUSE 3 [54]	EVT vs. no EVT based on perfusion imaging	6–16 h from symptom onset or last seen well LVO: ICA, M1-MCA NIHSS ≥ 6 ischemic core volume < 70 mL, mismatch ratio > 1.8, mismatch volume > 15 mL	ASPECT score < 6	ordinal score on mRS (90 d): EVT vs. control odds ratio, 2.77; *p* < 0.001	mRS 0–2: EVT 45% vs. control 17%, *p* < 0.001 Mortality: EVT 14% vs. control 26% *p* = 0.05 sICH: EVT 7% vs. control 4%, *p* = 0.75
DAWN [55]	EVT vs. no EVT based on MRI or perfusion imaging	6–24 h from symptom onset or last seen well LVO: ICA, M1-MCA NIHSS ≥ 10 0–<21 cc core infarct and NIHSS ≥ 10 (and age ≥ 80 years old) 0–<31 cc core infarct and NIHSS ≥ 10 (and age < 80 years old) 31 cc to < 51 cc core infarct and NIHSS ≥ 20 (and age < 80 years old)	involvement of >1/3 MCA territory	mean score on the utility-weighted mRS (90 d): EVT 5.5 vs. control 3.4 (adjusted difference 2.0 points; 95% credible interval, 1.1 to 3.0; posterior probability of superiority, >0.999 mRS 0–2 (90 d): EVT 49% vs. control 13% (adjusted difference, 33 percentage points; 95% credible interval, 24 to 44; posterior probability of superiority, >0.999)	sICH: EVT 6% vs. control 3% *p* = 0.50 mortality (90 d): EVT 19% vs. control 18%, *p* = 1.00)
RESCUE-Japan [58]	EVT vs. no EVT based on CT or MRI	last known well ≤ 6 h LVO: ICA, M1-MCA NIHSS ≥ 6 ASPECTS 3–5	Significant mass effect with midline shift on CT or MRI	mRS 0–3 (90 d): EVT 31.0% vs. control 12.7% (relative risk, 2.43; 95% CI, 1.35–4.37; *p* = 0.002)	improvement ≥ 8 NIHSS score at 48 h: EVT 31.0% vs. control 8.8% (relative risk, 3.51; 95% CI, 1.76–7.00) any ICH: EVT 58.0% vs. control 31.4%, (*p* < 0.001)
ANGEL-ASPECT [59]	EVT vs. no EVT based on CT or MRI or CTP	last known well ≤ 24 h LVO: ICA, M1-MCA NIHSS 6–30 ASPECTS 3–5 ASPECTS > 5 (6–24 h) with infarct core volume 70–100 mL ASPECTS < 3 with infarct core volume 70–100 mL	Midline shift, herniation or mass effect with effacement of the ventricles	shift in the distribution of the mRS scores (90d): generalized odds ratio, 1.37; 95% CI, 1.11–1.69; *p* = −2 0.004	mRS 0–2 (90d): EVT 30.0% vs. control 11.6% (relative risk, 2.62; 95% CI, 1.69–4.06) sICH: EVT 6.1% vs. control 2.7% (relative risk, 2.07; 95% CI, 0.79–5.41; *p* = 0.12)
SELECT2 [60]	EVT vs. no EVT based on CT or MRI or CTP	last known well ≤ 24 h LVO: ICA, M1-MCA NIHSS ≥ 6 ASPECTS 3–5 CT perfusion (rCBF < 30% ≥ 50 cc) MRI-DWI (ADC < 620 ≥ 50 cc)	significant mass effect with midline shift	shift in the distribution of mRS (90 d): EVT 4 (3–6) vs. control 5 (4–6), generalized odds ratio 1.51 (95% CI, 1.20–1.89; *p* < 0.001	mRS 0–2 (90 d): EVT 20% vs. control 7% (relative risk, 2.97; 95% CI, 1.60–5.51) sICH: EVT 0.6% vs. control 1.1% (relative risk, 0.49; 95% CI, 0.04–5.36)
TENSION [61]	EVT vs. no EVT based on CT or MRI	last known well ≤ 12 h LVO: ICA, M1-MCA NIHSS < 26 ASPECTS 3–5	Mass effect	shift in the distribution of mRS (90 d): EVT 4 (3–6) vs. control 6 (4–6), adjusted common OR 2.58, 95% CI, 1.60–4.15, *p* = 0.0001	mortality (90 d): hazard ratio 0.67, 95% CI, 0.46–0.98, *p* = 0.038 sICH: EVT 5% vs. control 5%
LASTE [62]	EVT vs. no EVT based on CT or MRI	last known well ≤ 6.5 h LVO: ICA, M1/M2-MCA NIHSS > 6 ASPECTS ≤ 5 ≥80 years old, ASPECTS 3–5	Significant mass effect with midline shift	shift in the distribution of mRS (90 d): EVT 4 (3–6) vs. control 6 (4–6), generalized odds ratio, 1.63; 95% CI, 1.29–2.06; *p* < 0.001	mRS 0–2 (90 d): EVT 13.3% vs. control 4.9% (adjusted relative risk, 2.39; 95% CI, 1.18–6.22) Mortality (90): EVT 36.1% vs. control 55.5% (adjusted relative risk, 0.65; 95% CI, 0.50–0.84) sICH: EVT 9.6% vs. control 5.7% (adjusted relative risk, 1.73; 95% CI, 0.78–4.68)
TESLA [63]	EVT vs. no EVT based on CT	last known well ≤ 24 h LVO: ICA, M1-MCA NIHSS > 6 ASPECTS 2–5	Midline shift or herniation	Improvement in 90 d functional outcome measured using mean utility-weighted mRS scores: mean EVT 2.93 (3.39) vs. control 2.27 (2.98), adjusted difference 0.63 (95% credible interval, −0.09 to 1.34; posterior probability for superiority of EVT, 0.96	Mortality (90 d): EVT 35.3% vs. control 33.3% sICH: EVT 4% vs. control 1.3%, Risk Ratio 2.96 (0.61 to 14.43)
DISTAL [66]	EVT vs. no EVT	last known well ≤ 6 h or 6–24 h with mismatch (>50%) Distal M2/M3/M4-MCA, A1/A2/A3-ACA, P1/P2/P3-PCA NIHSS 4–24, or NIHSS 2–24 with aphasia and/or hemianopia CTP or DWI/FLAIR mismatch 6–24 h	No tandem occlusion	Distribution of mRS (90 d): common odds ratio for improvement, 0.90; 95% CI, 0.67–1.22; *p* = 0.50	Mortality: EVT 15.5% vs. BMT 14.0% sICH: EVT 5.9% vs. BMT 2.6%
ESCAPE-MeVO [67]	EVT vs. no EVT based on CTA, CTP or MRI	last known well < 12 h Distal M2/M3-MCA, A2/A3-ACA, P2/P3-PCA NIHSS > 5	ASPECTS < 8 absence of collaterals in the affected territory core/penumbra mismatch	mRS 0–1 (90 d): EVT 41.6% vs. 43.1% BMT (adjusted rate ratio, 0.95; 95% CI, 0.79–1.15; *p* = 0.61	Mortality (90 d): EVT 13.3% vs. BMT 8.4% (adjusted hazard ratio, 1.82; 95% CI, 1.06–3.12). sICH: EVT 5.4%) vs. BMT 2.2%
DISCOUNT [68]	EVT vs. no EVT	last known well ≤ 6 h or ≤24 h if no hyperintense signal on FLAIR Distal M2/M3-MCA, A1/A2/A3-ACA, P1/P2/P3-PCA NIHSS ≥ 5	No tandem occlusion	mRS 0–2 (90 d): EVT 60% vs. 77% BMT (OR, 0.42; 95% CI, 0.2–0.88; *p* = 0.029)	Mortality: EVT 3% vs. BMT 7% sICH: EVT 12% vs. BMT 6%
EXPERTS [72]	ALT vs. standard treatment for posterior circulation stroke	Stroke onset 4–24 h pc-ASPECTS ≥ 7 NIHSS ≥ 1	Anterior circulation stroke	mRS 0–2 (90 d): ALT 89.6% vs. control 72.6%, (adjusted risk ratio, 1.16; 95% CI, 1.03–1.30; *p* = 0.01)	sICH: ALT 1.7% vs. control 0.9% (unadjusted risk ratio, 1.98; 95% CI, 0.18–21.56) Mortality (90 d): ALT 5.2% vs. control 8.5% (unadjusted risk ratio, 0.61; 95% CI, 0.23–1.62)
BEST [80]	EVT vs. BMT	Stroke onset < 8 h BA occlusion		mRS 0–3 (90 d): intention-to-treat EVT 42% vs. BMT 32%, (adjusted OR 1.74, 95% CI, 0.81–3.74)	mRS 0–3 (90 d): as-treated EVT 47% vs. BMT 24%, (adjusted OR, 3.02, 95% CI, 1.31–7.00) mortality (90 d): EVT 33% vs. BMT 38%, *p* = 0·54
BASICS [81]	EVT vs. BMT	Stroke onset < 6 h BA occlusion	Significant cerebellar mass effect or acute hydrocephalus Bilateral extended brainstem ischemia.	mRS 0–3 (90 d): EVT 44.2% vs. BMT 37.7%, (risk ratio, 1.18; 95% CI, 0.92–1.50)	sICH: EVT 4.5% vs. BMT 0.7% (risk ratio, 6.9; 95% CI, 0.9–53.0) mortality (90 d): EVT 38.3% vs. control 43.2%, (risk ratio, 0.87; 95% CI, 0.68–1.12)
BAOCHE [83]	EVT vs. BMT	Stroke onset 6–24 h BA occlusion or intracranial segments of both vertebral arteries NIHSS ≥ 6 Age 18–80	pc-ASPECTS < 6 Pons-midbrain-index ≥ 3 on CTA Complete cerebellar infarct on CT or MRI with significant mass effect and compression of the fourth ventricle Complete unilateral or bilateral thalamic infarction	mRS 0–3 (90 d): EVT 46% vs. BMT 24%, (adjusted rate ratio, 1.81; 95% CI, 1.26–2.6, *p* < 0.001)	sICH: EVT 6% vs. BMT 1%, (risk ratio, 5.18; 95% CI, 0.64–42.18) Mortality (90 d): EVT 31% vs. BMT 42% (adjusted risk ratio, 0.75; 95% CI, 0.54–1.04)
ATTENTION [84]	EVT vs. BMT	Stroke onset ≤ 12 BA occlusion NIHSS ≥ 10	pc-ASPECTS < 6 for patients < 80 years and <8 for patients ≥ 80 years cerebellar infarction with space occupying effect and compression of the fourth ventricle bilateral thalami or bilateral brainstem infarction	mRS 0–3 (90 d): EVT 46% vs. BMT 23%, (adjusted rate ratio, 2.06; 95% CI, 1.46–2.91, *p* < 0.001)	sICH: EVT 5% vs. BMT 0% Mortality (90 d): EVT 37% vs. BMT 55% (adjusted risk ratio, 0.66; 95% CI, 0.52–0.82)

ACA: anterior cerebral artery; ALT: alteplase; ASPECTS: Alberta Stroke Program Early CT Score; BA: basilar artery; BMT: best medical threatment; CBF: cerebral blood flow; CTA: CT angiography; CTP: CT perfusion; DWI: diffusion weighted imaging; EVT: endovascular treatment; FLAIR: fluid-attenuated inversion recovery; ICA: internal carotid artery; IVT: intravenous thrombolysis; LVO: large vessel occlusion; MCA: Middle cerebral artery; MRA: MR angiography; MRP: MR perfusion; mRS: modified Rankin Score; NCCT: non-contrast CT; NIHSS: National Institute of Health Stroke Scale; OR: odds ratio; PCA: posterior cerebral artery; pc-ASPECTS: posterior circulation ASPECTS; RCT: randomized controlled study; TNK: tenecteplase; TIA: transient ischemic attack; VB: vertebrobasilar.

**Table 3:** Ongoing randomized-controlled clinical trials (RCTs) of acute reperfusion treatments (IVT) in the extended time window.

Trial ID (Name)	Country	Status	Time from Symptom Onset	Intervention	Control	NIHSS	Age	Key Exclusion Criteria	Imaging
NCT06010628 (EXIT-BT2)	China	Recruiting	4.5–6 h	IV tenecteplase	Standard stroke care	≥4	>18	Planned IVT based on WAKE-UP or EXTEND criteria	NCCT
NCT06559436 (TNK-MeVO)	China	Recruiting	4.5–24 h	IV tenecteplase	Standard stroke care	≥4	>18	Planned EVT	DMVO (M1–M4, A1–A4, P1–P4), <50% core in vascular territory on NCCT, DWI-MRI, or CTP (>6 h)
NCT05199662 (RESILIENT EXTEND-IV)	Brazil	Recruiting	4.5–12 h	IV tenecteplase	Placebo	≥4 or cortical neurological deficit	>18	Proximal arterial occlusion (+ dominant M2 > 50% MCA territory)	CT/MRI with <50% territory involvement OR Core < 50 cc (NCCT/CTP/DWI), Mismatch Vol > 10 cc, Ratio > 1.4
NCT05752916 (OPTION)	China	Recruiting	4.5–24 h	IV tenecteplase	Antiplatelet agents	6–25	>18	Proximal LVO	Core < 50 cc, mismatch ratio ≥ 1.2, mismatch volume ≥ 10 cc
NCT04454788 (ETERNAL-LVO)	Australia	Terminated	<24 h	IV tenecteplase + EVT	EVT ± IV alteplase	Non-minor symptoms	>18	Basilar artery occlusion	LVO, CTP/DWI mismatch: Core < 70 mL, penumbra > 20 mL, ratio > 1.8
NCT05105633 (POST-ETERNAL)	Australia	Recruiting	<24 h	IV tenecteplase ± EVT	IV alteplase or no IVT ± EVT	Not reported	>18	Extensive brainstem ischemia or frank hypodensity on NCCT	pc-ASPECTS ≥ 7, Basilar artery occlusion
NCT07094763	China	Not yet recruiting	4.5–24 h	IV tenecteplase	Standard medical therapy	≥3	>18	Planning EVT	MRI-confirmed posterior circulation infarct OR hypoperfusion OR occlusion of posterior circulation vessel
BI 1123-0060 TENACITY NCT non available	International	Not yet recruiting	>4.5 h	IV tenecteplase	placebo	Disabling stroke	-	-	Salvageable ischemic tissue/perfusion imaging

CTP = computed tomography perfusion; DMVO = distal medium vessel occlusion; DWI-MRI = diffusion-weighted magnetic resonance imaging; EVT = endovascular treatment; IVT = intravenous thrombolysis; LVO = large vessel occlusion; MCA = middle cerebral artery; NCCT = non-contrast CT; NIHSS = National Institutes of Health Stroke Scale; pc-ASPECTS = posterior circulation Alberta Stroke Program Early CT Score.

**Table 4:** Ongoing randomized-controlled clinical trials (RCT) of acute reperfusion treatments (EVT) in the extended time window from ClinicalTrials.gov.

RCT	NCT06654375	NCT06560203 (BAOCHE2)	NCT05326932 (LATE-MT)	SELECT-LATE NCT Non-Available
**Country**	China	China	China	International (US, Europe, Australia)
**Status**	recruiting	recruiting	recruiting	Not yet recruiting
**Time from symptom onset**	24–72 h	24–72 h	24–72 h	24–72 h
**Intervention**	EVT	EVT	EVT	EVT
**Control**	best medical management	standard stroke care	standard medical care	best medical management
**NIHSS**	≥2	≥6	≥6	≥6
**Age**	>18	>18 and ≤80	>18	18–85
**Key exclusion criteria**	treated with alteplase > 4.5 h after last known well	eligible for IVT pc-ASPECTS) < 6 and Pons-midbrain-index of ≥3 on CT/CTA or MRI Complete bilateral thalamic infarction	EVT attempted after stroke onset	Eligible for thrombectomy or medical management
**Imaging**	ICA or MCA-M1 occlusion moderate to good collateral flow	Occlusion of the basilar artery or intracranial segments of both vertebral arteries	ICA or MCA-M1/M2 occlusion infarct core volume < 50 mL, mismatch ratio ≥ 1.8 and mismatch volume ≥ 15 m on CTP or MRP	ICA or MCA-M1/M2

CTP: computed tomography perfusion; EVT: MCA: middle cerebral artery; MRP: magnetic resonance imaging perfusion; NIHSS: National Institutes of Health Stroke Scale; pc-ASPECTS: posterior circulation Alberta Stroke Program Early CT Score.
